# The Increasing Role of Kappa Free Light Chains in the Diagnosis of Multiple Sclerosis

**DOI:** 10.3390/cells10113056

**Published:** 2021-11-06

**Authors:** Franz Felix Konen, Philipp Schwenkenbecher, Konstantin Fritz Jendretzky, Stefan Gingele, Kurt-Wolfram Sühs, Hayrettin Tumani, Marie Süße, Thomas Skripuletz

**Affiliations:** 1 Department of Neurology, Hannover Medical School, 30625 Hannover, Germany; konen.felix@mh-hannover.de (F.F.K.); schwenkenbecher.philipp@mh-hannover.de (P.S.); jendretzky.konstantin@mh-hannover.de (K.F.J.); gingele.stefan@mh-hannover.de (S.G.); suehs.kurt-wolfram@mh-hannover.de (K.-W.S.); 2 Department of Neurology, University of Ulm, 89081 Ulm, Germany; hayrettin.tumani@uni-ulm.de; 3 Department of Neurology, University Medicine Greifswald, 17475 Greifswald, Germany; marie.suesse@uni-greifswald.de

**Keywords:** multiple sclerosis, kappa free light chains, cerebrospinal fluid, serum, biomarker, review

## Abstract

Free light chains (FLC) are a promising biomarker to detect intrathecal inflammation in patients with inflammatory central nervous system (CNS) diseases, including multiple sclerosis (MS). The diagnostic use of this biomarker, in particular the kappa isoform of FLC (“KFLC”), has been investigated for more than 40 years. Based on an extensive literature review, we found that an agreement on the correct method for evaluating KFLC concentrations has not yet been reached. KFLC indices with varying cut-off values and blood-CSF-barrier (Q_Albumin_) related non-linear formulas for KFLC interpretation have been investigated in several studies. All approaches revealed high diagnostic sensitivity and specificity compared with the oligoclonal bands, which are considered the gold standard for the detection of intrathecally synthesized immunoglobulins. Measurement of KFLC is fully automated, rater-independent, and has been shown to be stable against most pre-analytic influencing factors. In conclusion, the determination of KFLC represents a promising diagnostic approach to show intrathecal inflammation in neuroinflammatory diseases. Multicenter studies are needed to show the diagnostic sensitivity and specificity of KFLC in MS by using the latest McDonald criteria and appropriate, as well as standardized, cut-off values for KFLC concentrations, preferably considering non-linear formulas such as Reiber’s diagram.

## 1. Introduction

As early as the 1970s, radial immunodiffusion and radioimmunoassays were used to detect free light chains (FLC) [1]. Based on the fact that FLC are parts of intact immunoglobulins, it was hypothesized that they might be a biomarker for inflammatory activity [1]. FLC are produced by B and plasma cells and exist in two isoforms, kappa and lambda [2,3]. In 1980, Stendahl-Brodin and colleagues investigated the concentration of kappa free light chains (KFLC) in serum and cerebrospinal fluid (CSF) of patients suffering from multiple sclerosis (MS) using a nephelometric assay [4]. Since this time, many studies have investigated the diagnostic value of FLC in patients with intrathecal inflammation, mostly in patients with MS, as this is one of the most common neurologic autoimmune diseases with strong intrathecal inflammation [5]. To date, no single available biomarker has had sufficient specificity to detect MS. Multiple proposals of diagnostic criteria have been made over time to differentiate between definite and probable MS and clinically isolated syndrome (CIS). A timely diagnosis and initiation of therapy are of great importance to slow down disease progression [6,7,8,9,10,11,12,13,14,15,16]. The latest diagnostic criteria for MS are based on evidence of dissemination of inflammation in space and time in the central nervous system (CNS) [16]. Applying these revised McDonald criteria for MS from 2017, the proof of an intrathecal inflammation in CSF can substitute as a criterion for dissemination in time [16]. Currently, oligoclonal bands (OCB) constitute the gold standard for detection of an intrathecal inflammation, which are isoelectrically focused and subsequently stained immunoglobulin G (IgG) antibodies in CSF [17]. KFLC are promising additional biomarkers to detect intrathecal inflammation with a similar performance in comparison with OCB analytics [18,19,20,21,22,23]. The determination of KFLC in CSF and serum has been performed fully automated for several years and the most common measurement methods are nephelometric, turbidimetric, and ELISA-assays [18,19]. The advantages of automated measurements are rapid results, less expensive diagnostics, and rater-independency [20,21,22,23]. However, various challenges, including the lack of standard reference values, have prevented KFLC from being implemented into daily clinical routine so far.

Most frequently, FLC measurements were interpreted by using different threshold values of the KFLC index (CSF/serum KFLC quotient (Q_KFLC_)/CSF/serum albumin quotient (Q_Albumin_)) or different Q_Albumin_-based diagrams [24]. In 2019, Reiber and colleagues developed a quotient diagram based on a hyperbolic function to establish a reference range for KFLC in CSF similar to the hyperbolic function for the immunoglobulins A, G, and M [25]. This reference range has already been successfully applied in different studies and showed a superior performance in terms of diagnostic sensitivity for the detection of intrathecal inflammation as compared with other reference values for KFLC and the gold standard OCB [24,25,26,27,28,29]. However, the role of KFLC in other neurologic diseases than MS is not entirely clear. Because KFLC are non-specific markers of inflammation in the CNS, it is reasonable to assume that they are also produced intrathecally in other infectious or inflammatory CNS diseases. In this review, our goal was to provide a comprehensive summary of previous research on KFLCs as diagnostic biomarkers in inflammatory CNS diseases.

## 2. Materials and Methods

The literature search was performed using the PubMed database. General search terms were used in order to obtain the largest number of publications. As shown in Supplemental Figure S1, 243 articles were found within a period from 1975 to 2021. All cited publications were manually screened for content concerning MS and KFLC. Of these publications, 119 were considered for this review article. In addition, 69 publications were identified that determined OCB and KFLC concentrations in CSF and serum of patients suffering from MS or CIS, which were included in the analysis of different threshold values of KFLC.

## 3. Results

### 3.1. Different Determination Methods of OCB

OCB constitute the current gold standard to detect an intrathecal IgG synthesis. For this reason, they have been included in the latest revision of the McDonald criteria of 2017 as substitute for the fulfillment of dissemination in time [16,17]. When examining the sensitivity of OCB analysis, it is important to keep in mind that there are different methods to determine OCB.

In all methods for detection of OCB, the highest diagnostic sensitivity is reached by a separation of type G immunoglobulins in CSF and serum using isoelectric focusing [17,30,31]. IgG of different molecular weights and charges can be separated by a pH- and an electric gradient, while other immunoglobulin types cannot be separated [17,32]. Silver staining, immunofixation, and immunoblotting are different techniques to visualize the separated immunoglobulin bands. For immunofixation and immunoblotting anti-human immunoglobulins that bind the Fc-part of the separated IgG are added, followed by a peroxidase staining to make bands visible [33,34]. For silver staining, polyacrylamide gels are impregnated with silver ions, which are consecutively reduced to metallic silver [35,36,37]. The autocatalytic growth of the deposit of insoluble metallic silver in the sample is initiated by an initial deposit of insoluble silver leading to the visualization of the sample [35,36,37]. Some studies indicated that the most sensitive method for the determination of intrathecal IgG might be the visualization of isoelectrically focused OCB by silver staining [38,39,40,41]. Studies using isoelectric focusing and consecutive silver staining in polyacrylamide gels for the determination of OCB reached a diagnostic sensitivity of about 99% in MS patients, which was superior to KFLC measurement [24,27,42]. However, a similar procedure for OCB determination conducted at a different laboratory site reached a diagnostic sensitivity of 89% in MS patients, indicating the dependency of conductor and rater of non-automated determination methods [43]. Studies included in this review used immunofixation, immunoblotting, or Western blotting assays for OCB determination. These studies reached a diagnostic sensitivity between 78 and 98% when OCB positivity was not an inclusion criterion [4,18,19,20,21,22,23,25,26,28,29,33,44,45,46,47,48,49,50,51,52,53,54,55,56,57,58,59,60,61,62,63,64,65,66,67,68,69,70,71,72,73,74,75,76,77,78,79,80,81,82,83,84,85,86,87,88,89,90,91,92]. Thus, in the majority of these studies, KFLC measurement was superior to OCB determination [4,18,19,20,21,22,23,25,26,28,29,33,44,45,46,47,48,49,50,51,52,53,54,55,56,57,58,59,60,61,62,63,64,65,66,67,68,69,70,71,72,73,74,75,76,77,78,79,80,81,82,83,84,85,86,87,88,89,90,91,92]. To date, there is no comparative study investigating which OCB method is the most sensitive. Nevertheless, most studies using polyacrylamide gels and silver staining to detect OCB showed a better performance with higher rates of OCB-positive patients [24,27,42].

### 3.2. Kappa and Lambda Free Light Chains

An intact immunoglobulin molecule consists of two identical heavy chains and two covalently bound light chains. The synthesis of heavy and light chains and the immunoglobulin assembly take place in B and plasma cells [2,3]. Light chains are formed by different fragments and are required for antibody activity as they contribute to the antigen-binding site [2,3]. Although light chains are produced in excess even in healthy patients, only a low concentration of unbound, so-called free light chains (FLC), can be detected [2,3]. Light chains occur in two isoforms, the kappa FLC (KFLC), which are mostly found in a monomeric form, and lambda FLC (LFLC), which are more often observed in a dimeric form [2,3]. There is evidence that changes in structure and level of dimeric or polymeric FLC may contribute to the pathogenesis of primary systemic amyloidosis [2,3]. In addition, different studies suggest that FLC may have important functions and elicit mast cell-mediated hypersensitivity reactions, which are part of asthma and contact sensitivity [93,94]. Accordingly, an FLC antagonist was shown to potently inhibit the development of contact sensitivity, asthma, and inflammatory bowel diseases in animal models [93,94].

The major catabolic site of light chains is the kidneys [2,3]. Several types of endopeptidases and proteolytic factors cleave light chains into the variable and the constant region while the variable region is not further degradable under physiologic conditions [2,3]. Since the kidneys provide light chain proteolysis and excretion via urine, abnormally elevated light chain concentrations primarily damage the kidneys [2,3]. Increased serum light chain concentrations may be the result of two pathophysiologically different mechanisms: enhanced synthesis and reduced clearance [2,3]. Enhanced synthesis may be found in different disease entities (e.g., monoclonal gammopathies, primary systemic amyloidosis, systemic lupus erythematosus), while a reduced clearance may be a consequence of these disorders or due to changes within the kidney itself (e.g., age-related) [2,3]. Nevertheless, independent of the origin of elevated serum light chain concentrations, different mechanisms may mediate injury of the kidneys, subsequently leading to a reduction of renal function [2,3].

KFLC and LFLC are detectable by nephelometry, turbidimetry, and ELISA [18,19]. In MS and CIS patients, the sensitivity of KFLC to detect intrathecal Ig synthesis is superior to LFLC, which has been demonstrated in several studies [4,19,23,45,46,47,48,49,50,51,52,53,54,55,56,57,58,59,60,61,62,63,64,65,66,67,68,69,87]. The mean diagnostic sensitivity and specificity of KFLC measurement in MS patients was 90% in each (sensitivity 24–100%; specificity 68–100%), while LFLC measurement revealed a mean sensitivity of 69% (9–100%) and a specificity of 84% (46–100%) [4,19,23,45,46,47,48,49,50,51,52,53,54,55,56,57,58,59,60,61,62,63,64,65,66,67,68,69,87]. Nevertheless, it has to be taken into consideration that some studies investigated threshold values for KFLC and LFLC endeavoring for a maximum of diagnostic sensitivity or specificity. In addition, different evaluation methods for cut-off values were chosen, leading to the reported wide range of these parameters. In almost all studies, the results of KFLC measurement revealed a better correlation with OCB than LFLC [4,19,23,45,46,47,48,49,50,51,52,53,54,55,56,57,58,59,60,61,62,63,64,65,66,67,68,69,87]. Huss and colleagues showed that a positive MRZ-reaction (positive antibody specific indices (AI) for at least two viruses of the following: measles-, rubella- and varicella-zoster-virus) was associated with higher KFLC values (CSF concentration, Q_KFLC_, KFLC index), while a significant correlation between LFLC and positive MRZ-reaction was not observed [47]. Similar results were shown by Süße and colleagues: according to the Reiber’s diagram for KFLC, patients with a positive MRZ reaction revealed higher intrathecally produced KFLC concentrations than MRZ-negative patients [25,28]. Therefore, KFLC measurement is favored in comparison with LFLC in the diagnosis of MS, which is mirrored in the more common use of KFLC-based threshold values like the KFLC index or Q_KFLC_ and Q_Albumin_-dependent diagrams [24,25].

### 3.3. Comparability of Common KFLC Methods

Few studies investigated the comparability of KFLC determination by the most common measurement methods: nephelometry, turbidimetry, and ELISA [19,61,64,80]. Süße and colleagues showed that Siemens (N Latex FLC kappa kit, Siemens Healthcare Diagnostics Products GmbH, Marburg, Germany) and The Binding Site (Freelite, The Binding Site Ltd., Birmingham, UK) assays were similar for KFLC measurement in CSF and serum [80]. Significant differences between both nephelometric assays were not observed [80]. Applying a KFLC index of 3.61 as threshold value, a diagnostic sensitivity of 100% and a specificity of 88% was reached [80]. Similar results were shown in the study of Bernardi and colleagues [64]. Nephelometric assays of Siemens (N Latex FLC kappa kit, Siemens Healthcare Diagnostics Products GmbH, Marburg, Germany) and The Binding Site (Freelite, The Binding Site Ltd., Birmingham, UK) reached similar diagnostic sensitivity (Siemens 100%, The Binding Site 95%), while the determination of KFLC was superior to LFLC measurement (Siemens 93%, The Binding Site 83%) [64]. Zeman et al. compared a turbidimetric assay (Freelite Kappa SPA_PLUS_ kit and Freelite Lambda SPA_PLUS_ kit, The Binding Site Ltd., Birmingham, UK), a nephelometric assay (N Latex FLC kit, Siemens Healthcare Diagnostics Products GmbH, Marburg, Germany), a commercially available ELISA (Human Immunoglobulin Free Light Chains Kappa and Lambda ELISA kit, BioVendor-Laboratorni medicina a.s., Brno, Czech Republic), and an in-house ELISA, which revealed similar results [19]. Receiver operating curve (ROC) analysis showed an area under the curve of 81–91% for all assays including the measurement of LFLC regarding the differentiation of MS and non-MS patients [19]. Makshakov and colleagues compared a nephelometric assay (which was not further specified) with an ELISA-assay based on monoclonal anti-kappa and anti-lambda antibodies directed against cryptic epitopes of free FLC molecules (Polignost Ltd., St. Petersburg, Russia), which revealed better results than the nephelometric assay according to the authors [61]. Interestingly, Bayart et al., Zeman et al., and Sindic et al. investigated the utility of determination of FLC by isoelectric focusing [19,78,95]. Sindic and colleagues found a good correlation between FLC detection using a quantitative method (affinity-mediated capillary blotting) and a qualitative method (particle-counting immunoassay as described by Fagnart and colleagues) in MS patients [95,96]. Bayart and colleagues proposed that isoelectrically focused KFLC might be useful when OCB testing is negative [78]. Ten out of fourteen OCB-negative MS patients revealed KFLC bands in the CSF while 38% of OCB-negative patients (MS and inflammatory neurological disease controls) with KFLC bands in CSF were KFLC index negative (cut-off 6.29; Freelite Kappa SPA_PLUS_ kit, The Binding Site Ltd., Birmingham, UK). This indicates the complementarity of quantitative and qualitative measurement methods [78]. Zeman et al. showed the value of oligoclonal KFLC as well: 24% of the patients who were positive for oligoclonal KFLC testing were OCB-negative [19].

### 3.4. Alternative KFLC Determination Methods

The determination of KFLC by Western blotting is not recommended by the authors since the comparability with the most common measurement methods (nephelometry, turbidimetry, or ELISA) has not yet been investigated. Ganelin-Cohen et al. and Kaplan et al. used an SDS-PAGE-based Western blot assay to analyze CSF and serum samples [97,98,99]. The intensity of KFLC and LFLC immunoreactive bands was evaluated by a self-developed software as a measure of KFLC and LFLC concentrations [97,98,99]. Different threshold values for FLC concentrations in CSF, CSF-serum concentration quotients, monomer and dimer levels and indices of CSF-serum quotients of MS patients were compared with controls, achieving a diagnostic sensitivity of 91–100% in MS patients and 67% in CIS patients [97,98,99].

Radial immunodiffusion, radioimmunoassays, or particle-counting immunoassays as qualitative determination methods for KFLC were used in 2006 by Rinker and colleagues and were the measurement methods of choice in the time from 1970 to 2000 [1,96,100,101,102]. Other qualitative tests such as immunoelectrophoresis and electroimmunofixation were also used in the same time period by different authors [95,103,104,105,106,107,108,109,110]. However, these studies reported very heterogeneous results for the diagnostic accuracy of KFLC and LFLC in patients with MS [1,95,96,100,101,102,103,104,105,106,107,108,109,110]. In addition, the authors often investigated how the experiments had to be conducted, leading to the publication of protocols for the respective determination method rather than to the publication of diagnostic sensitivity and specificity of the respective test [1,95,96,100,101,102,103,104,105,106,107,108,109,110]. Since radial immunodiffusion, radioimmunoassays, particle-counting immunoassays, and immunoelectrophoresis are no longer frequently used, these studies were not included in the analysis of different threshold values of KFLC and the comparison with OCB in the present review article [1,95,96,100,101,102,103,104,105,106,107,108,109,110].

### 3.5. FLC Investigations in Body Fluids Other Than Serum and CSF

Few studies investigated the diagnostic utility of FLC in other human body fluids like urine [63,111,112,113,114,115]. These studies revealed that FLC could be found more frequently in the urine of patients with MS than in healthy controls or other non-inflammatory diseases, especially when relapses occurred [63,111,112,113,114,115]. Since urine FLC originates from blood, elevated urine FLC concentrations theoretically indicate high serum FLC concentrations, which are subsequently excreted, or renal dysfunction with relevant proteinuria. However, neither elevated serum FLC concentrations nor renal function impairment are known to be characteristic for MS patients, thus undermining the diagnostic utility of urine FLC in MS. Further relevant problems in the usage of body fluids other than CSF and serum include missing pre-analytic standards and thus missing consequences for the patient in the clinical routine at that moment. In addition, there is no consent for which isoform of FLC is of greater diagnostic utility: kappa or lambda [63,111,112,113,114,115]. Furthermore, a wide range in the concentration of FLC in urine has been observed, which indicates a limited diagnostic value and seems to be applicable only for patients with very high concentrations [111,112]. Due to the wide fluctuation of KFLC in urine, only a poor correlation between urine KFLC concentrations and clinical disease activity as expressed by Kurtzke’s expanded disability status scale (EDSS) and brain magnetic resonance imaging (MRI) was found [112,114].

Totolian et al. investigated the concentrations of KFLC and LFLC in lacrimal fluid and saliva and found increased concentrations of KFLC compared with the controls [115]. Lotan et al. and Kaplan et al. determined monomer and dimer isoforms of FLC in saliva using a Western blot assay [116,117]. Both studies revealed that calculated saliva FLC index values might be a useful biomarker to differentiate between a healthy state and active MS, with a sensitivity of about 90% [116,117]. Conclusively, the authors emphasized that the analysis of non-invasively gained body fluids could be of importance in the biochemical follow-up of patients or the therapeutic monitoring as the inflammatory disease activity and immunosuppressive treatment with corticosteroids influenced the concentration of KFLC in urine and saliva [63,111,112,113,114,115,116,117]. However, theoretical considerations and the low number of reports undermine the role of FLC in body fluids other than CSF and serum in the diagnostic work-up of MS patients.

### 3.6. Influencing Factors for FLC and Impact on Laboratory Analysis Procedure

Before pre-analytic influencing factors are considered, it generally has to be kept in mind that the detection of intrathecally synthesized KFLC does not only mirror the presence of an IgG synthesis in CNS, but also of IgA and IgM [25,118]. A summary of investigated influencing factors on the concentrations of KFLC is given in Table 1.

Various pre-analytic impact factors of KFLC were investigated, of them blood contamination of CSF. Since the contamination of CSF with blood displays a passive passage of molecules during the sample collection, it should not be mistaken for a real de novo synthesis within the brain. On the one hand, it could be shown that blood contamination of CSF with volume amounts up to 20,000 erythrocytes did not influence the results of KFLC measurements in CSF [27]. On the other hand, Hannich et al. investigated the influence of blood contamination on Reiber’s diagram for KFLC [25,118]. The extent of blood contamination of CSF and the concomitant passive passage of immunoglobulins was sufficient to display an artificial intrathecal immunoglobulin synthesis according to Reiber’s diagrams for IgG, IgA, and IgM [118]. In contrast, the same extent of blood contamination of CSF did not lead to an artificial intrathecal synthesis according to Reiber’s diagram for KFLC [25,118]. Thus, the authors concluded that the interpretation of Reiber’s diagram for KFLC was stable against the influence of blood contamination of CSF [25,118]. This is of great relevance since the concentrations of intact immunoglobulins are influenced by blood contamination. Depending on the molecular size of the immunoglobulins (IgM > IgA > IgG), only very low concentrations of IgM followed by IgA and IgG are found in CSF [119]. In line with this assumption, it was previously shown that the influence of blood contamination on CSF results was higher for IgM, followed by IgA and IgG [120]. An explanation might be that blood contamination of CSF samples bypasses the selectivity of the blood–CSF barrier, which is usually molecular size-related [118]. Therefore, instead of following a hyperbolic reference range, CSF-serum quotients of KFLC and immunoglobulins follow a more linear function, which is either situated below (immunoglobulin quotients) or above (Q_KFLC_) the linear function [118]. Subsequently, due to a blood contamination of CSF samples, immunoglobulin and KFLC quotients approach the bisecting line resulting either in approaching (immunoglobulin quotients) or distancing from the upper reference line [118].

Storage duration by either room temperature or 4 °C for two weeks did not lead to significant alterations in the concentration of KFLC in CSF or serum [27]. The usage of serum or EDTA tubes had no influence on the concentration of KFLC either [27]. In addition, different therapeutic procedures, including apheresis with plasma exchange or immunoadsorption and the application of intravenous immunoglobulins, did not influence the amount of KFLC in serum [27]. In contrast, the infusion of 2000 mg of intravenous methylprednisolone led to a significant reduction of KFLC in serum, which proceeded with further infusions of methylprednisolone [27]. This is a remarkable finding since there is no significant alteration in concentrations of IgG, IgA, and IgM in serum, and both proteins, immunoglobulins, and KFLC share the same cellular origin. A possible explanation might be that reduction of B cell activity and subsequent lower KFLC synthesis combined with rapid renal excretion (half-life time in vivo 2–6 h) lowers the actual serum KFLC concentrations [121,122,123,124]. The reduced serum KFLC concentration, in combination with unaltered CSF concentrations, might lead to a falsification of Q_KFLC_-based interpretation tools, such as the KFLC index or Reiber’s diagram for KFLC [27]. As the KFLC concentration in serum was reduced independently of the age, sex, and disease of the patient, a decrease of the serum KFLC concentration of about 28% after the first 1000 mg of intravenous methylprednisolone, 40% after 2000 mg, and 49% after 5000 mg has to be kept in mind [27].

In contrast, intrathecal application of rituximab significantly increased the amount of KFLC in CSF in comparison with the baseline concentration, although the authors denoted this effect as “minimal” [125]. An alteration of KFLC concentrations in serum and LFLC concentrations (CSF and serum) was not shown [125]. The investigation of KFLC under therapy with interferons (IFNβ-1a) showed stable concentrations in CSF over a two-year time course [77]. Rosenstein et al. could not observe significant changes of KFLC indices in patients treated with fingolimod or alemtuzumab when baseline levels and values after 12 or 24 months were compared [43].

Furthermore, different studies investigated patient-related impact factors on the concentration of KFLC and whether or not a significant correlation between patient sex, age and KFLC concentrations might exist [18,50,71,72,74,78,81]. In some studies, a correlation between increasing patient age and decreasing KFLC indices was observed with higher indices in female patients [18,71,81]. Pieri et al. investigated gender-specific KFLC index threshold-values and found a cut-off of 12.5 for women and 11 for men [81]. In contrast, other studies did not report a significant correlation [50,72,74,78].

Nevertheless, a recent study systematically investigated the influence of rising patient age and renal dysfunction on KFLC [126]. The physiologically observed slower CSF flow with increasing age displayed by the usage of age-dependent Q_Albumin_ thresholds had an influence on CSF KFLC concentrations [126,127]. Furthermore, the concentration of KFLC in serum was increased in patients with renal dysfunction [128,129]. It was shown that decreasing renal function led to declining KFLC indices and KFLC IF, according to Reiber’s diagram for KFLC [25,126]. In addition, in patient samples with an “inflammatory” CSF profile, 15% of the patients presented a KFLC index <5.9 while 10% of these patients showed an intrathecal KFLC fraction below Q_KFLC_(lim), suggesting possible false-negative KFLC results [25,126]. The authors concluded that the influence of renal function should be considered while interpreting KFLC results in patients with neuroinflammatory diseases [126]. Furthermore, Reiber’s diagram for KFLC seemed to be less susceptible to alterations due to renal function impairment and should therefore be favored when interpreting KFLC concentrations [25,126].

**Table 1:** Influence factors on kappa free light chain (KFLC) concentrations.

Reference	Investigated Pre-Analytic Impact Factor	Influence on KFLC Concentration
[126]	patient-related (renal dysfunction, age)	lower KFLC indices and KFLC IF in renal dysfunction; more false negative results using KFLC index
[18,50,71,72,74,78,81]	patient-related (sex)	not entirely clear (suggestion of higher concentration in females)
[27,118]	blood contamination of CSF	no influence on CSF KFLC concentrations (up to 20,000 erythrocytes/mL CSF); no influence on Reiber’s diagram for KFLC (Reiber et al., 2019, Ref. No. 25)
[27]	storage duration and temperature usage of EDTA or serum tubes	no influence (up to 14 days by either room temperatur or 4 °C); no influence (usage of EDTA or serum tube)
[27]	acute first-line therapy (intravenous methylprednisolone) acute second-line therapy (plasmapheresis, immunoadsorption, intravenous immunoglobulins)	decrease of serum KFLC concentrations (28% after 1000 mg, 40% after 2000 mg, 49% after 5000 mg) no influence
[43,77]	disease-modifying therapy (interferon β-1a, fingolimod, alemtuzumab)	no influence
[125]	experimental therapy (intrathecal rituximab)	significant increase of CSF KFLC concentrations (no influence on serum concentrations)

Shown are investigated pre-analytic influencing factors on KFLC concentrations in cerebrospinal fluid (CSF) and serum, as well as interpretation methods like KFLC indices and intrathecal KFLC fractions, according to Reiber’s diagram for KFLC (KFLC IF) [25]. Further, reference numbers are given.

### 3.7. KFLC Threshold Values in the Diagnosis of MS

The results from many studies evaluating the diagnostic accuracy of KFLC with different study designs, populations, and index tests are summarized in this paragraph. Comparison and summarization of these studies is complicated since the study design strongly affects results interpretation. Nevertheless, knowing these limitations, the present approach was chosen and 95% confidence intervals were given to provide an overview of reported diagnostic accuracies. Three different approaches were suggested to interpret FLC concentrations in order to diagnose MS. The findings are summarized in Table 2.

Some studies, which investigated the utility of FLC measurement in the diagnosis of MS, empirically created cut-off values either for the absolute KFLC or LFLC concentrations in CSF, CSF/serum KFLC, and LFLC quotients (Q_KFLC_, Q_LFLC_), CSF KFLC/IgG, and LFLC/IgG ratios or KFLC/LFLC concentration ratios to determine KFLC or LFLC positivity [4,19,20,23,33,47,48,50,52,53,54,55,60,61,62,63,64,65,66,67,68,73,74,76,77,85,88,89,90,91,130]. The cut-off for the CSF KFLC concentration varied between 0.103 µg/mL and 7 mg/L and a mean diagnostic sensitivity and specificity of 86% (95% confidence interval (80%, 92%)) and 91% (86%, 96%) was reached while the threshold for CSF LFLC concentration varied between 0.0175 µg/mL and 4.3 mg/L and a mean sensitivity and specificity of 67% (48%, 85%) and 82% (67%, 96%) was reported [4,19,20,23,33,47,48,50,52,53,54,55,60,61,62,63,64,65,66,67,68,73,74,76,77,85,88,89,90,91,130]. The cut-off for Q_KFLC_ was set between 4.9 and 30 leading to a mean diagnostic sensitivity of 88% (79%, 96%) and specificity of 90% (77%, 100%) compared with the cut-off for Q_LFLC_ (1.9 and 12.5), which reached a mean sensitivity of 33% (min–max: 21–45%) and specificity of 90% (min–max: 88–92%) [4,19,20,23,33,47,48,50,52,53,54,55,60,61,62,63,64,65,66,67,68,73,74,76,77,85,88,89,90,91,130]. Vecchio et al. also investigated the utility of CSF KFLC/IgG and LFLC/IgG ratios and reached diagnostic sensitivity of 87% and 51% and specificity of 88% each [68]. Investigating KFLC/LFLC concentration quotients, Stendahl-Brodin et al. set a ratio of >1:7 as cut-off and reached a diagnostic sensitivity of 45% in MS patients while Vecchio et al. found a sensitivity and specificity of 78% each applying a different threshold value [4,68]. In the study of Rathbone et al., which investigated the KFLC/LFLC ratio in CSF too, an elevated ratio in MS patients was observed [52].

Problematically, these studies did not take the influence of the blood–CSF barrier function into account. Without consideration of Q_Albumin_ as a marker for the blood–CSF barrier function, absolute CSF FLC concentrations or KFLC/LFLC ratios hazard the risk of false-positive results. In addition, the inter-individual variation of FLC concentrations due to the patient’s age was ignored.

In the last years, the most common approach has been the implementation of Q_Albumin_ and the proposal of an FLC index. The index calculation may be employed for the evaluation of intrathecal synthesis of KFLC as well as for LFLC. Different studies applied KFLC indices varying between 0.92 and 20 and reached a mean diagnostic sensitivity of 87% (82%, 92%) and a mean diagnostic specificity of 87% in MS patients (83%, 91%) [19,20,21,22,23,24,27,28,29,33,42,44,45,46,47,48,49,50,51,53,54,56,57,58,59,60,61,64,65,68,69,70,71,72,73,74,75,78,79,80,81,82,85,87,92]. It has to be considered that in some of these studies, the diagnostic utility of FLC in OCB-negative MS patients was investigated, leading to very low diagnostic sensitivity. Some studies also investigated LFLC indices (cut-off 0.29–21.45) leading to lower mean sensitivity (64% (46%, 81%)) and specificity (83% (74%, 93%)) [19,20,21,22,23,24,27,28,29,33,42,44,45,46,47,48,49,50,51,53,54,56,57,58,59,60,61,64,65,68,69,70,71,72,73,74,75,78,79,80,81,82,85,87,92]. FLC indices implement Q_Albumin_ and therefore are more accurate in detecting the intrathecally synthesized portion and differentiating it from FLC, which diffused from blood into CSF.

Problematically, linear cut-off values do not integrate the non-linear diffusion of blood-derived proteins of different sizes over an intact blood–CSF barrier, leading to false positive and false negative results as already observed for total IgG [131]. In addition, proposed KFLC and LFLC indices vary considerably and a consensus about a consistent KFLC or LFLC index is not in sight. Therefore, the most physiologic approach was the constitution of Q_Albumin_-dependent, mostly non-linear functions to determine a cut-off, which is in accordance with the laws of diffusion [25,51,65,83,84,88].

Presslauer et al.’s non-linear formula was applied in different studies and reached a mean diagnostic sensitivity and specificity of 91% each (sensitivity (78%, 100%), specificity (69%, 100%)) [18,20,23,24,33,50,56,74,84]. The proposed function of Senel and colleagues reached a mean diagnostic sensitivity of 95% (78%, 100%) and a specificity of 94% [24,51]. Already in 2014, Senel et al. had established an empirically determined linear threshold line, which detected 87% of all investigated MS patients [83]. In 2004 and 2009, Arneth et al. and Fischer et al. used Q_Albumin_-dependent threshold lines to investigate FLC positivity in MS patients and reached absolute diagnostic sensitivity (100%) [65,88]. The latest proposed threshold line was Reiber’s diagram for KFLC [25]. In the first comparative study of this kind, OCB determined by the silver staining method was compared with the most common KFLC index of 5.9 and the latest Q_Albumin_-dependent functions of Presslauer et al., Senel et al., and Reiber et al. [24,25,51,66,84]. In a cohort consisting of 100 MS patients, 24 CIS patients with conversion to MS and 44 CIS patients without conversion to MS, the diagnostic superiority of Reiber’s diagram for KFLC was shown [24]. Reiber’s diagram was applied in further studies and a diagnostic sensitivity of 97% (94%, 99%) and specificity of 75% (53%, 97%) was reported [24,25,26,27,28,29,43,68,75]. Further, Süße et al. used Reiber’s diagram to determine a cut-off value based on the intrathecal fraction of KFLC (KFLC IF according to Reiber’s diagram) in order to differentiate between neuromyelitis optica spectrum disorders (NMOSD) and MS [25,26]. Summarizing, the authors recommend the application of Reiber’s diagram for KFLC for the interpretation of KFLC concentrations.

**Table 2:** Kappa free light chain (KFLC) threshold values in the diagnosis of MS.

Reference	Algorithm Used for KFLC Calculation	Diagnostic Sensitivity (CI)	Diagnostic Specificity (CI)	*p*-Value
[24,25,26,27,28,29,43,68,75]	Reiber’s diagram for KFLC: Q_Kappa_(lim) = (3.27 (Q_Alb_^2^ + 33)^0.5^ − 8.2) × 10^−3^	97% (94%, 99%)	75% (53%, 97%)	<0.0001
[18,20,23,24,33,50,56,74,84]	Presslauer’s non-linear function: KFLC_Lim_ = 0.9358 × Q_Alb_^0.6687^	91% (78%, 100%)	91% (69%, 100%)	<0.0001
[24,51]	Senel’s linear function (2019): Q_KFLC_ = 14.85 + 2.41 × Q_Alb_	95% (78%, 100%)	94% (92%, 95%)	0.0091
[19,20,21,22,23,24,27,28,29,33,42,44,45,46,47,48,49,50,51,53,54,56,57,58,59,60,61,64,65,68,69,70,71,72,73,74,75,78,79,80,81,82,85,87,92]	KFLC index *: Q_KFLC_/Q_Alb_	87% (82%, 92%)	87% (83%, 91%)	<0.0001
[4,19,20,23,33,47,48,50,52,53,54,55,60,61,62,63,64,65,66,67,68,73,74,76,77,85,88,89,90,91,130]	KFLC quotient **: CSF KFLC/serum KFLC	88% (79%, 96%)	90% (77%, 100%)	<0.0001
[4,19,20,23,33,47,48,50,52,53,54,55,60,61,62,63,64,65,66,67,68,73,74,76,77,85,88,89,90,91,130]	CSF KFLC concentration ***	86% (80%, 92%)	91% (86%, 96%)	<0.0001
* threshold varying between 0.92 and 20; ** threshold varying between 4.9 and 30; *** threshold varying between 0.103 µg/L and 7 mg/L				

Shown are different KFLC threshold values for the diagnosis of MS. The reference number as well as the algorithm used for KFLC calculation are given. Further, diagnostic sensitivity and specificity, 95% confidence interval (CI) and *p*-value are shown. Cerebrospinal fluid is denoted “CSF”, KFLC quotient “Q_KFLC_”, and the albumin quotient “Q_Alb_”.

### 3.8. The Value of KFLC as a CSF Biomarker in the Diagnosis of MS

The McDonald criteria allow diagnosing MS, based solely on MRI when dissemination of CNS lesions in both space and time can be demonstrated [16]. On the other hand, according to the revised criteria of 2017, dissemination in time might be substituted by the presence of OCB in CSF [16]. It has already been reported by different authors that by the implementation of OCB as a substitute for dissemination in time, MS can be diagnosed more frequently at the time of the first clinical event as compared to the previous McDonald criteria [16,132]. Furthermore, OCB positivity in CIS patients is an important indicator for the later diagnosis of MS [9,133,134]. Because KFLC has similar analytic sensitivity to detect intrathecal inflammation as OCB the question arises whether they have the same diagnostic and prognostic value.

Thus, studies, which applied the revised McDonald criteria of 2017 in order to evaluate the performance of KFLC in the diagnosis of MS and CIS, constitute the most relevant investigations for this review (Table 3 and Table 4) [16]. Over all studies, the mean prevalence of OCB positivity in MS patients was 94% (min–max: 85–100%) reflecting the importance of detection of intrathecal inflammation as a criterion for dissemination in time [24,26,27,28,29,33,42,43,44,47,48,55,56,68,69,70,71,72,73,74,75,92]. Nevertheless, it has to be considered that some studies only included OCB-positive MS patients while other studies comprised a certain percentage of OCB-negative MS patients, thus leading the observed wide range of OCB prevalence [24,26,27,28,29,33,42,43,44,47,48,55,56,68,69,70,71,72,73,74,75,92]. KFLC measurements, interpreted by different approaches, reached a mean diagnostic sensitivity of 94% (min–max: 72–100%) and specificity of 84% (min–max: 65–100%) in MS patients [24,26,27,28,29,33,42,43,44,47,48,55,56,68,69,70,71,72,73,74,75,92]. Patients with CIS, including patients with and without the later diagnosis of MS during follow up revealed an OCB prevalence of 78% (25–100%) [24,26,27,28,29,33,42,47,48,55,56,69,70,71,72,73,74,75]. The diagnostic sensitivity of KFLC in these patients varied between 17% and 100% while data for the diagnostic specificity was not available [24,26,27,28,29,33,42,47,48,55,56,69,70,71,72,73,74,75]. The low diagnostic sensitivity and the wide range in CIS patients of both OCB and KFLC might be caused by the influence of the latest, more inclusive McDonald criteria, the missing differentiation of CIS patients with and without conversion to MS, and due to the usage of different cut-off methods [16,132]. However, when applying the 2017 McDonald criteria, it has to be taken into consideration that MS or CIS patients might present with atypical clinical manifestations and misleading MRI findings [16,132]. In these cases, additional evidence of intrathecal inflammation may be helpful in making the correct diagnosis. In the application of OCB or KFLC, one should always be aware that these biomarkers are not specific for a particular disease but are expressions of non-specific intrathecal immunoglobulin synthesis. Thus, the CSF findings should be carefully interpreted in context with clinical presentation and MRI findings of the patient [132].

### 3.9. Correlation of FLC with Brain MRI and Disability Progression in MS

Results are heterogeneous concerning the correlation of KFLC concentrations or calculated KFLC measurements like quotients or indices with MRI parameters of inflammation of the CNS. In some studies, a significant correlation between elevated KFLC concentrations in CSF or KFLC indices and brain atrophy, brain lesion pattern, or T2-lesion volume was observed [45,69,135]. In contrast, other studies have not shown a significant correlation between KFLC and brain damage according to MRI or localization and load of MRI abnormalities [43,56,62,66,72,76,77].

Similarly, the prediction of disease and disability progression based on KFLC concentrations was evaluated heterogeneously. Some studies revealed a significant correlation between high KFLC concentrations in CSF and early disability or disability progression mostly estimated by the EDSS [61,67,72,76,81,98,130]. In contrast, Rathbone and colleagues observed that a high CSF KFLC/LFLC ratio was associated with a lower median EDSS [52]. Berek and colleagues reported that a high KFLC index (>100) at baseline was associated with a shorter time to clinically definite MS and with a higher chance for a second clinical attack within 24 months from baseline [69]. In contrast, other studies denied a correlation between disease progression evaluated by EDSS and KFLC CSF concentration or KFLC indices [43,66,69,77,84,87]. In some studies, high KFLC CSF concentrations or KFLC indices were associated with a higher risk for CIS patients to be later diagnosed with definite MS [43,50,53,54,61,68,69,73,82,83,86,130]. In other studies, this correlation could not have been observed, and CIS patients with conversion to MS did not reveal elevated CSF KFLC [55,84,87].

The heterogeneous results concerning the correlation between CSF KFLC concentrations or indices and MRI findings, disease and disability progression and the rates of CIS conversion to MS might be partly caused by the application of different diagnostic criteria for MS, different interpretations methods and analyzed parameters. In addition, most studies were performed retrospectively, which hazard the risk of a selection bias for the inclusion of patients. Furthermore, the reported results might be influenced by the inclusion of MS patients with different disease duration and disease-related disabilities, the limited number of investigated patients, or the short follow-up time [43,45,62,66,69,72,76,77,135]. Thus, heterogeneity of the reported results of different studies and limited comparability might be expected.

### 3.10. Outlook

The determination of KFLC is a promising marker to show intrathecal inflammation in the diagnostic process of patients with MS. Multicenter studies are needed to introduce non-linear formulas for KFLC as the new standard to determine the threshold for intrathecally produced KFLC. Considering the multiple pre-analytical and analytical influencing factors on KFLC concentrations that need to be corrected for, Reiber’s diagram for KFLC should be used preferably. To date, such studies concerning KFLC in the diagnosis of MS according to the 2017 revision of the McDonald criteria are lacking. In addition, only a few studies that applied the revised McDonald criteria of 2017 chose a prospective study design, thus, most investigations hazard the risk of a selection bias [29,33,55,69,70,74,75,92]. To further investigate the role of KFLC as a prognostic biomarker in CIS patients, studies with longer follow-up duration are needed. The mean follow-up time of the studies included in this review was only 35 months (min–max: 24–58 months) [43,50,53,55,58,61,68,69,73,82,83,84,86,87,130]. Furthermore, the knowledge about KFLC in other neurologic diseases with intrathecal inflammation is rare. Several studies used patients with inflammatory or infectious neurologic diseases as control groups for the comparison with MS patients [29,44,45,46,51,69,70,71,75,78]. Apart from that, there is one study, which systematically investigated KFLC concentrations in patients suffering from a viral CNS infection (tickborne-encephalitis) [136]. In this study, significant differences of CSF and serum KFLC concentrations before and after treatment were reported [136]. Two studies investigated KFLC concentrations in samples of neuroborreliosis patients and found the determination of KFLC to be highly sensitive and specific [137,138]. Nevertheless, further studies need to investigate KFLC in different viral and bacterial infections of the CNS and the influence of treatment in these disease entities. This knowledge is of great importance as a biochemical differentiation through CSF analysis might be possible. OCB positivity is not a common finding at the time of the first diagnostic lumbar puncture in patients with bacterial meningitis [139,140]. One might speculate that KFLC are superior to OCB in detecting intrathecal inflammation in infectious CNS diseases at an early time point in the disease course because they represent not only IgG but also IgA and IgM. This hypothesis is supported by the empirical work of Hannich et al., which showed that KFLC synthesis is detectable not only in the context of intrathecal IgG but also in the presence of intrathecal IgA and IgM synthesis [118]. Furthermore, a quantitative measurement of KFLC might detect a new active intrathecal inflammation or a decrease of inflammatory activity, and thus may be a superior biomarker to monitor disease activity compared with OCB [26,141]. To date, there are only a few studies that have systematically investigated KFLC in NMOSD, an autoimmune disease, which can mimic MS [26,71,75]. In concordance with predominantly negative OCB, Süße et al. and Cavalla et al. revealed significantly lower KFLC concentrations and intrathecal fractions in patients with NMOSD in comparison with MS patients [26,71]. Other diseases, such as autoimmune encephalitis, which were part of the “other neuro-inflammatory diseases” control group, revealed similar KFLC concentrations as NMOSD and were therefore significantly lower than in MS patients [71]. Further, Ferraro and colleagues showed that the KFLC index has a diagnostic accuracy in the prediction of an inflammatory (including NMOSD) or infectious CNS disease compared with OCB [75]. Nevertheless, the value of KFLC measurement in autoimmune encephalitis patients, which are characterized by the presence of intrathecal IgG antibodies directed against different neuronal structures, have not been systematically investigated to date.

## 4. Conclusions

Determination of KFLC has emerged as a promising biomarker for intrathecal inflammation in patients with MS in over 40 years of investigation. The most common and recommended measurement methods include nephelometry, turbidimetry, and ELISA assays, which seem to be equivalent and comparable to each other. Measurement of KFLC in other body fluids than serum and CSF (urine, saliva, lacrimal fluid) is not recommended for therapeutic monitoring or biochemical follow-up of MS patients. KFLC are highly prevalent in MS patients, who are diagnosed according to the revised criteria of 2017. However, large multicenter studies investigating MS and CIS patients with longer follow-up duration are still missing. Furthermore, KFLC determination has been rarely investigated in infectious diseases of the CNS and autoimmune disorders, which can mimic MS. Lastly, the lack of a consistent interpretation method prevented KFLC determination from being implemented in the diagnostic routine work-up. For a reliable interpretation of KFLC concentrations in CSF and serum sample pairs, the usage of Reiber’s diagram is recommended in consideration of its pathophysiologically based formula, its low influenceability against pre-analytic factors, and its outstanding diagnostic sensitivity.

## Figures and Tables

**Table 3:** Kappa free light chain (KFLC) determination in multiple sclerosis (MS) patients according to the revised McDonald criteria of 2017.

Reference	*N* MS Patients	OCB Determination (Gel for IEF)	KFLC Determination	OCB Positivity in MS (%)	KFLC Positivity in MS (%)
[43]	223	silver-staining (polyacrylamide)	nephelometry *	88%	Reiber’s diagram for KFLC: 96%
[26]	26	immunofixation (agarose)	nephelometry *	89%	Reiber’s diagram for KFLC: 96%
[27]	82	silver-staining (polyacrylamide)	nephelometry *	99%	Reiber’s diagram for KFLC: 98%
[28]	68	immunofixation (agarose)	nephelometry *	94%	Reiber’s diagram for KFLC: 100%
[29]	3 selected MS patients (with one band in CSF)	immunofixation (agarose)	nephelometry *	NA	Reiber’s diagram for KFLC: 100%
[75]	84	immunoblotting (agarose)	turbidimetry **	85%	Reiber’s diagram for KFLC: 92%
[68]	133	immunoblotting (agarose)	nephelometry *	96%	Reiber’s diagram for KFLC: 98%
[23]	100	silver-staining (polyacrylamide)	nephelometry *	99%	Reiber’s diagram for KFLC: 98%
[33]	104	immunoblotting (agarose)	nephelometry *	87%	KFLC IF (Presslauer’s curve) > −0.41: 95%
[69]	38	immunoblotting (polyacrylamide)	nephelometry *	95%	KFLC index > 6.6: 86%
[44]	34	immunofixation (agarose)	turbidimetry ***	100%	KFLC index > 9.417: 94%
[70]	39	immunofixation (agarose)	turbidimetry **	94%	KFLC index > 2.9: 97%
[48]	68 selected MS patients (38 OCB+, 30 OCB-)	immunofixation (agarose)	nephelometry **	NA	KFLC index > 3.09: 72%
[71]	130	immunofixation (agarose)	nephelometry *	86%	KFLC index > 6.15: 90%
[72]	100 (initial relapse-remitting MS course 84)	immunofixation (agarose)	nephelometry *	92%	KFLC index > 5.0: 92%
[74]	127	immunofixation (agarose)	nephelometry *	97%	KFLC index > 5.0: 96%
[42]	83	silver-staining (polyacrylamide)	nephelometry *	99%	KFLC index > 5.9: 95%
[56]	70 selected MS patients (40 OCB+, 30 OCB-)	immunofixation (NA)	nephelometry **	NA	KFLC index > 4.25: 94%
[73]	50	immunofixation (agarose)	nephelometry *	100%	KFLC index > 9.092: 90%
[92]	45	immunofixation (agarose)	turbidimetry **	89%	KFLC index > 3.045: 98%
[55]	41 (according to 2010 or 2017 McDonald criteria)	NA	turbidimetry **	93%	CSF KFLC > 7 mg/L: 95%
[47]	65	immunoblotting (polyacrylamide)	nephelometry *	97%	NA
* *N* Latex FLC kappa assay (Siemens Healthcare Diagnostics Products GmbH, Marburg, Germany), ** Freelite assay (The Binding Site Ltd., Birmingham, UK), *** Optilite assay (The Binding Site Ltd., Birmingham, UK)					

Studies (reference number) are shown, which determined KFLC and cerebrospinal fluid (CSF)-specific oligoclonal bands (OCB) of MS patients diagnosed according to the revised McDonald criteria of 2017. Furthermore, the quantity of investigated MS patients (*N*) and the sensitivity of OCB and KFLC values are shown. In addition, the OCB determination method and the respective gel, which was used for isoelectric focusing (IEF), as well as the KFLC determination method and the appendant assay, are described. Oligoclonal band positivity was denoted “OCB+” and oligoclonal band negativity “OCB-”.

**Table 4:** Kappa free light chain (KFLC) determination in clinically isolated syndrome (CIS) patients according to the revised McDonald criteria of 2017.

Reference	*N* CIS Patients	OCB Positivity in CIS (%)	KFLC Positivity in CIS (%)
[28]	16 (CISMS), 9 (CISCIS), 5 lost to follow-up	93%	Reiber’s diagram for KFLC: 100%
[29]	4 (selected CIS patients with one band in CSF)	NA	Reiber’s diagram for KFLC: 100%
[24]	24 (CISMS), 44 (CISCIS)	88% (CISMS), 25% (CISCIS)	Reiber’s diagram for KFLC: 88% (CISMS), 20% (CISCIS)
[69]	38 (CISMS), 50 (CISCIS)	95% (CISMS), 86% (CISCIS)	KFLC indices CISMS > CISCIS
[43]	20	60%	KFLC index > 3.4: 75%; KFLC indices MS > CIS
[68]	6 (CISMS), 12 (CISCIS)	96%	KFLC indices CISMS > CISCIS
[42]	25 (CISMS), 41 (CISCIS)	80% (CISMS), 27% (CISCIS)	KFLC index > 5.9: 72% (CISMS), 17% (CISCIS)
[55]	22 (CISMS), 14 (CISCIS) (according to 2010 or 2017 McDonald criteria)	90%	KFLC indices CISMS > CISCIS (not significant)
[73]	50 (CISMS), 51 (CISCIS)	100%	KFLC indices CISMS > CISCIS
[33]	20	NA	NA
[70]	1 (included in “MS study population”)	NA	NA
[71]	10 (included in “MS study population”	NA	NA
[72]	11 (included in “MS study population”)	NA	NA
[92]	3	100%	NA
[75]	28	NA	NA

Studies (reference number) are shown, which determined KFLC and cerebrospinal fluid (CSF)-specific oligoclonal bands (OCB) of CIS patients diagnosed according to the revised McDonald criteria of 2017. Furthermore, the quantity of investigated CIS patients (*N*) and the sensitivity of OCB and KFLC values are shown. Oligoclonal band positivity was denoted “OCB+”, oligoclonal band negativity “OCB-”, CIS with conversion to multiple sclerosis (MS) “CISMS”, and CIS without conversion “CISCIS”.

## Data Availability

The datasets used and/or analyzed during the current study are available from the corresponding author on reasonable request.

## Supplementary Materials

The following are available online at https://www.mdpi.com/article/10.3390/cells10113056/s1, Figure S1. Literature search strategy.

## Abbreviations

FLC	free light chains
KFLC	kappa free light chains
LFLC	lambda free light chains
CSF	cerebrospinal fluid
Ig	immunoglobulin
MS	multiple sclerosis
CIS	clinically isolated syndrome
NMOSD	neuromyelitis optica spectrum disorders
ELISA	enzyme-linked immunosorbent assay
EDSS	expanded disability status scale
MRI	magnetic resonance imaging
EDTA	ethylenediaminetetraacetic acid
