# Supplementary material for: The Increasing Role of Kappa Free Light Chains in the Diagnosis of Multiple Sclerosis
Source: Cells. 2021 Nov 6;10(11):3056. doi: 10.3390/cells10113056 (PMC8622045; doi:10.3390/cells10113056)
Supplement: Supplementary file 1 [file cells-10-03056-s001.zip › Supplemental Figure S1.pdf]

cells

MDPI

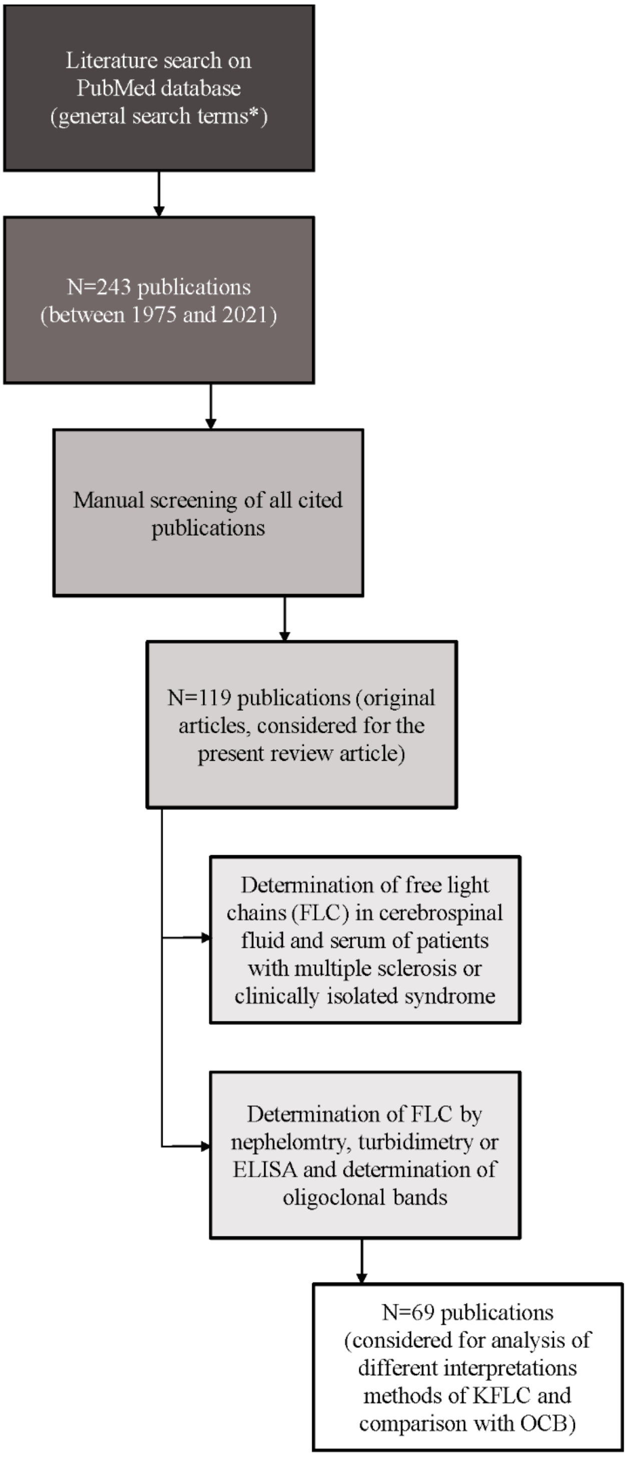

*General search terms: "free light chains multiple sclerosis", "free light chains MS", "FLC MS", "kappa free light chains multiple sclerosis", "KFLC multiple sclerosis", "KFLC MS", "kappa free light chains MS", "lambda free light chains multiple sclerosis", "LFLC multiple sclerosis", "LFLC MS", "lambda free light chains MS".

Supplemental Figure S1. Literature search strategy.
